# Natural Deep Eutectic Solvent-Based Matrix Solid Phase Dispersion (MSPD) Extraction for Determination of Bioactive Compounds from Sandy Everlasting (*Helichrysum arenarium* L.): A Case of Stability Study

**DOI:** 10.3390/plants11243468

**Published:** 2022-12-11

**Authors:** Milena Ivanović, Peter Krajnc, Aleš Mlinarič, Maša Islamčević Razboršek

**Affiliations:** 1 Faculty of Chemistry and Chemical Engineering, University of Maribor, Smetanova ulica 17, SI-2000 Maribor, Slovenia; 2 Marifarm, Proizvodnja in Storitve d.o.o., Minařikova ulica 8, SI-2000 Maribor, Slovenia

**Keywords:** matrix solid phase dispersion extraction (MSPD), natural deep eutectic solvents (NADES), extraction optimization, sandy everlasting, phenolic compounds, stability studies, antioxidant activity

## Abstract

In the present study, vortex-assisted matrix solid-phase dispersion (VA-MSPD) extraction was used to isolate the major bioactive compounds from *H. arenarium*. To reduce the negative environmental impact of the conventionally used organic solvents, four different choline chloride-based natural deep eutectic solvents (NADES) were investigated as possible eluents. The most influential VA-MSPD extraction parameters: stationary phase (adsorbent), adsorbent/sample ratio, vortex time, and volume of extraction solvent were systematically optimized. Ultrasound-assisted extraction with 80% MeOH was used as the standard method for the comparison of results. The stability of the obtained extracts was studied over a period of 0 to 60 days at three different temperatures (−18 °C, 4 °C, and 25 °C). All extracts were evaluated both spectrophotometrically (determination of total phenolic content (TPC) and antioxidant activity by ABTS and FRAP assay) and chromatographically (HPLC-UV). NADES based on choline chloride and lactic acid (ChCl-LA) was selected as the most effective extractant, with a determined TPC value of its extract of 38.34 ± 0.09 mg GA/g DW (27% higher than the methanolic VA-MSPD extract) and high antioxidant activity. The content of individual phenolic compounds (chlorogenic acid, dicaffeoylquinic acid isomers, naringenin isomers, and chalcones) in the ChCl-LA extract, determined by HPLC-UV, was comparable to that of the conventionally obtained one. Moreover, the stabilization effect of ChCl-LA was confirmed for the studied compounds: chlorogenic acid, naringenin-4′-O-glucoside, tomoroside A, naringenin-5-O-glucoside, isosalipurposide, and naringenin. The optimum VA-MSPD conditions for the extraction of *H. arenarium* polyphenols were: florisil/sample ratio of 0.5/1, a vortex time of 2 min, and an elution volume of ChCl-LA of 10 mL.

## 1. Introduction

Naturally occurring phenolic compounds (PC), which have been shown to have health-promoting effects, particularly preventive role in various diseases caused by oxidative stress, are important components of the human diet [1]. Moreover, the biological activities of PC make them interesting as natural antioxidants for many fields such as cosmetics, pharmaceuticals, food packaging, and the textile industry [2]. However, to make naturally occurring PC commercially useful, several difficulties need to be overcome, such as their extraction and concentration from plant material (their concentration in plant tissues is usually low); the evaluation of their stability and protection from various external factors (most of them are sensitive to influences such as high temperatures), solar radiation or humidity; and a deeper understanding of their actual biological effects and potential applications [2,3,4,5,6].

Between these factors, the development of new, faster, simpler, and more environmentally friendly extraction methods that simultaneously maximize the utilization of the process and prevent the degradation of the target compounds is certainly a challenge for both research and industrial sectors. Therefore, in addition to other well-described extraction techniques such as supercritical fluid extraction (SFE), ultrasound-assisted extraction (UAE), and microwave-assisted extraction (UAE), improved solid-phase extraction (SPE) methods such as QuEChERS (quick, easy, cheap, effective, rugged, and safe) [7], matrix solid-phase dispersion extraction [8,9], magnetic solid-phase extraction [10], have also been developed for the isolation of bioactive compounds from various plant matrices. Matrix solid-phase dispersion extraction (MSPD), first developed by Barker et al. [11], is a modified SPE in which the solid, semi-solid, or viscous sample is ground with some solid adsorbents before eluting the target compounds with the selected solvent under vacuum [12]. Recently, to simplify the method and improve the extraction efficiency, MSPD has been further enhanced by the use of different external factors such as electric field (E-MSPD) [13], magnetic field [14], ultrasound (UA-MSPD) [15,16], and vortex (VA-MSPD) [9,17,18,19]. VA-MSPD is certainly the most acceptable method because of its simplicity. The main feature of this method is that the elution step is replaced by the vortexing of the crushed sample and the dispersed phase with a solvent, which increases the contact area between the solvent and the target molecules [9,12,20].

The genus *Helichrysum*, with over 600 different species, including the well-known species of the genus *H. italicum*, *H. stoechas*, and *H. arenarium*, are traditionally used as ornamental plants, medicines, and dietary supplements due to their high content of various bioactive compounds with proven antioxidant, antimicrobial and anti-inflammatory activities [21,22,23,24,25]. *H. arenarium* is a rich source of several flavonoid classes but is the first to be recognized as a natural source of the flavanone naringenin and its isomers, including the chalcones isosalipurposide and tomoroside A [25,26,27]. Several lines of investigation suggest that therapeutic supplementation with naringenin is beneficial in the treatment of obesity, diabetes, hypertension, and metabolic syndrome [28,29,30]. Conventional solid–liquid extraction methods such as soxhlet extraction [22], maceration, MAE [31], and UAE [24] with the different solvents (methanol, ethanol, and ionic liquids) have been previously used for the extraction of bioactive compounds from the inflorescences of *H. arenarium*. In our recently published study, we demonstrated the applicability of natural deep eutectic solvents (NADES) as a promising green solvent for the isolation of major phenolic compounds from *H. arenarium* [26].

NADES are natural analogs of deep eutectic solvents (DES) formed by mixing hydrogen bond donors (HBD) and hydrogen bond acceptors (HBA) commonly found in nature, such as sugars, organic acids, polyalcohols, and others. Previous studies have shown that NADES fully meet the current requirements of green analytical chemistry [32], and their application in various fields of the same and especially in extraction processes are increasingly the subject of research by numerous research groups [4,6,26,33,34,35,36].

In this study, for the first time, the simplicity and effectiveness of VA-MSPD were combined with the environmental friendliness of NADES to develop a rapid, inexpensive, and green extraction method for the isolation of the valuable PC from the *H. arenarium*. All factors that could potentially affect the extraction process, such as the type and volume of extraction solvent (NADES), sample-to-adsorbent ratio (S/A ratio), sample-to-extraction solvent ratio, and extraction time, were further optimized using factor-per-time analysis. However, since recent studies have focused on the direct use of NADES extracts without further purification [35,36], this work also includes a stability study of PC in these solvents. Consequently, the stability of the major PC found in the inflorescences of *H. arenarium* (chlorogenic acid, naringenin, and its isomers, isosalipurposide and tomoroside A) was monitored over a period of up to two months.

## 2. Results and Discussion

### 2.1. Selection of the NADES for the VA-MSPD

The first part of the study focused on the selection of the most suitable NADES for the extraction of the major PC from *H. arenarium* using VA-MSPD. For that purpose, four different NADES based on choline chloride (HBA) and lactic acid (organic acid), 1,2-propanediol (polyalcohol), fructose (sugar), and urea (amide) as HBD were evaluated. All the extracts obtained were analyzed by two instrumental techniques: spectrophotometric (determination of TPC, mg GA/g DW) and chromatographic (determination of the content of the major PC, mg/10 g DW). Based on the available literature [23,24,25,26,27], as well as on the preliminary results of this study (Figure S1, supplementary material) it was confirmed that the most dominant PC in the inflorescence of *H. arenarium* are phenolic acids (chlorogenic acid and isomers of dicaffeolyquinic acid), flavonoids (naringenin and its glucosides, apigenin and its glucosides, and kaempferol glucosides) and chalcones (tomoroside A and isosalipurposide). Consequently, for VA-MSPD optimization, the content of six characteristic PC was monitored: chlorogenic acid (RR_T_ = 9.37 min), naringenin-4′-*O*-glucoside (Nar-4-gluc, RR_T_ = 14.45 min), tomoroside A (TomA, RR_T_ = 14.65 min), naringenin-5-*O*-glucoside (Nar-5-gluc, RR_T_ = 15.01 min), isosalipurposide (Iso, RR_T_ = 20.01) and naringenin (Nar, RR_T_ = 26.05 min). The results were compared (Table 1) with those obtained using 80% MeOH as extraction solvent by using both methods, VA-MSPD and the reference UAE method.

A comparison of the obtained results (Table 1) with our previous study suggests that, in most cases, the extraction efficiency of the tested NADES for the extraction of *H. arenarium* PC is determined by the chemical structure of the compounds and the NADES composition, while the extraction method affects the content of the extracted compounds to a lesser extent. Even in the case of 80% MeOH, no differences were observed when UAE and VA-MSPD were used as the extraction techniques. The only deviation from this trend was observed in the extraction of the chalcones (TomA and Iso), where significantly higher concentrations were found when VA-MSPD was applied. However, considering all the advantages of VA-MSPD over UAE, such as time, energy, and chemical savings, simplicity of application, lower investment cost, and reduced environmental impact, the development of this method for the extraction of bioactive compounds is certainly justified [9,19,37,38]. One of the first research studies that addressed the application of MSPD in combination with alternative green solvents (in this case ionic liquids) for the isolation of flavonoid glucosides from lime fruit was published in 2016 by Xu et al. [38]. They found that the optimal extraction conditions were as follows: Florisil (150 mg) as adsorbent (sample/adsorbent ratio 1:3), a grinding time of 1 min, and 1-butyl-3-methylimidazolium tetrafluoroborate (0.4 mL, 250 mM) as elution solvent. However, most researchers are increasingly skeptical about the environmental friendliness of ionic liquids [39], and the extraction trends are largely toward the use of DES and/or NADES as the extraction solvents. Consequently, in 2019, the first study by Wu et al. discussed the possibility of using MSPD in combination with DES for the determination of aflatoxins from crops [37], while the first studies demonstrating the possibility of PC extraction were published in 2021 [14,16]. The MSPD method was effectively applied using tetramethylammonium chloride:ethylene glycol (1:3) NADES as eluent for the extraction of naringenin, hesperidin, and gallic acid from the fruit juices [16]. On the other hand, the optimal DES for the magnetic-assisted MSPD (MA-MSPD) of flavonoids (morin, quercetin, and kaempferol) from different sources was tetramethylammonium chloride:lactic acid [14]. In our particular case, based on the preliminary results (Table 1), a choline chloride:lactic acid NADES (ChCl-LA) was selected for further optimization of the method.

### 2.2. Optimization of NADES-VA-MSPD Method

After the selection of the extraction solvent, the proposed NADES-VA-MSPD method was further optimized with respect to the following influencing parameters: (a) selection of the stationary phase (adsorbent), (b) ratio of sample to adsorbent (S/A ratio), (c) time of vortex, and (d) ratio of sample to extraction solvent. In this part of the study, in addition to the determination of TPC and concentration of target compounds, antioxidant activity was determined for all extracts obtained using two standard spectrophotometric methods (ABTS and FRAP).

#### 2.2.1. Selection of the Stationary Phase (Adsorbent)

**Figure 1:**
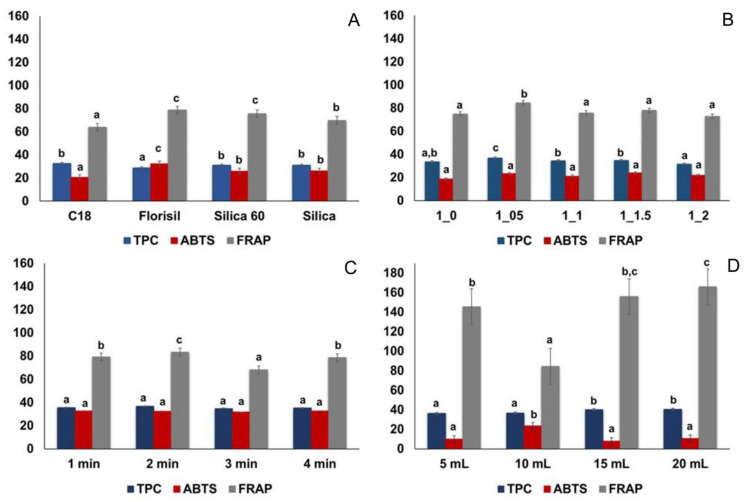
Influence of extraction parameters: (**A**) selection of stationary phase (adsorbent); (**B**) ratio of sample to adsorbent (S/A ratio); (**C**) time of vortexing and (**D**) ratio of sample to extraction solvent on the: TPC (mg GA/g DW); ABTS (mg TE/g DW) and FRAP (mg Fe^2+^/g DW) for the *H. arenarium* extracts obtained by the NADES-VA-MSPD extraction method. Different superscripts for the same variable indicate a statistically significant difference at the 95% confidence level (*p* < 0.05) according to the S-N-K test.

**Figure 2:**
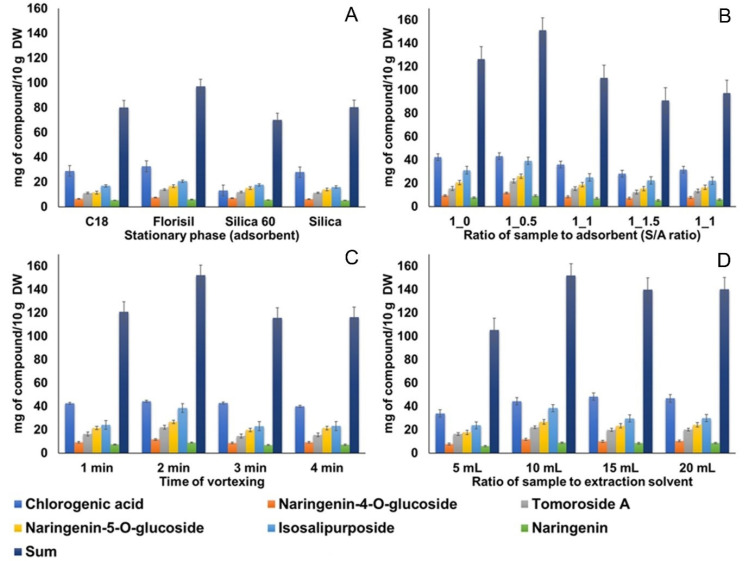
Influence of extraction parameters: (**A**) stationary phase selection (adsorbent); (**B**) ratio of sample to adsorbent (S/A ratio); (**C**) time of vortexing and (**D**) ratio of sample to extraction solvent on the extraction yield of individual phenolic compounds (mg/10 g DW) from *H. arenarium* inflorescences by NADES-VA-MSPD.

In the development and optimization of MSPD methods, the selection of the appropriate adsorbent is a crucial step. The role of the stationary phase in the process is not only to break up and disperse the sample particles, but also to interact with the target compounds and separate them based on the strength of their interaction [8,40]. In this work, four commercially available and inexpensive stationary phases (Florisil, C18, and two silicas) were evaluated. The results obtained are shown graphically in Figure 1A and Figure 2A.

In the case of TPC, the highest value (32.67 ± 0.07 mg GA/g DW) was determined in the extract using C18 as the adsorbent, but no statistically significant difference was confirmed between this result and those obtained with two silicas as the stationary phase (Figure 1A). The antioxidant activity (determined by ABTS) was significantly higher in the extract obtained with Florisil stationary phase (Figure 1A), while in the FRAP assay the statistically equal results were obtained for the extracts when Florisil and silica 60 were used as adsorbents. Although most previous studies suggest the use of C18 for MSPD extraction of individual PC from the plant sources [8,9,40], in our particular case, the highest contents were determined in the extracts with the stationary phase Florisil (Figure 2A). This could be due to the fact that the extraction capacity of the solid adsorbent is strongly influenced by the chemical structures of the target molecules and their mutual interactions. Florisil is a magnesium-bonded silicate gel that, like silica, has a polar structure and is compatible with polar PC molecules, while C18 has a higher affinity for less polar molecules due to its nonpolar structure [41]. The use of Florisil as the best stationary phase for the extraction of highly polar flavonoid glucosides has also been previously confirmed [38]. The results of our study also show that the size of silica particles does not affect the extraction process of the target PC, since no significant differences were observed between the contents, except in the case of chlorogenic acid, where silica 60 provided a significantly higher value (31.14 ± 0.14 mg/10 g DW) compared to silica (27.95 ± 0.14 mg/10 g DW) (Figure 2A). Considering all the results obtained, Florisil was selected as the stationary phase with the best performance for the desired application.

#### 2.2.2. Determination of the Optimal Sample-to-Adsorbent Ratio (S/A Ratio)

After the selection of the adsorbent (Florisil), different S/A (w:w) ratios (1:0.5, 1:1, 1:1.5, and 1:2) were evaluated (Figure 1B and Figure 2B). For comparison purposes, extraction without the addition of Florisil was also performed. The experimental results (Figure 2B) showed that the content of individual PC increased significantly with the increase in the S/A ratio from 1:0 to 1:0.5. This result was in good agreement with the recent results published by Peng et al. [42] and Nooraee Nia and Reza Hadjmohammadi [16]. On the other hand, some other authors have demonstrated the S/A ratio of 1:2 [40,41], 1:3 [38], or even 1:4 [43] as optimal. However, in our study, increasing the mass of the stationary phase beyond 1:0.5 resulted in a significant downward trend in extraction efficiency. This phenomenon can probably be explained by the fact that a larger amount of adsorbent leads to stronger molecular interactions such as hydrogen bonding and electrostatic forces between analytes and adsorbents [8], resulting in lower elution of target compounds from the adsorbent surface due to the higher viscosity of NADES compared to the conventionally used organic solvents. A similar trend was also observed in the results obtained by spectrophotometric methods (Figure 1B). Indeed, the statistically highest values for TPC (36.70 ± 0.20 mgGA/g DW) and FRAP (84.45 ± 1.12 mg Fe^2+^/g DW) were obtained in the extract with an S/A ratio of 1:0.5, while no differences were found in the results of the ABTS test. Consequently, an S/A ratio of 1:0.5 was selected for further optimization.

#### 2.2.3. Optimization of Vortex Time

MSPD assisted by vortex can adequately elute the analytes from the dispersion sorbent. In this study, vortex time from 1 min to 4 min was investigated [9,17]. From Figure 1C it can be concluded that increasing the vortex time does not lead to significant changes in the results for TPC and ABTS antioxidant assay, while the highest FRAP value (83.42 ± 2.01 mg Fe^2+^/g DW) was determined in the extract obtained after 2 min of vortex. For the individual PC, no significant effect on extraction efficiency was observed for chlorogenic acid and naringenin aglicone, while for the other compounds with a more complex structures (naringenin glucosides, TomA and Iso), the vortex time of 2 min was confirmed to be optimal (Figure 2C). These results agreed well with recent studies by Chen et al. [20] and Yan et al. [19], and the vortex time of 2 min was retained for further optimization.

#### 2.2.4. Optimization of Sample-to-Solvent Volume Ratio

The influence of the volume of the eluent in the extraction processes (including VA-MSPD) is significant, as it can affect the elution of the desired compounds from the surface of the adsorbent and consequently the final concentration of the active compounds in the obtained extracts [9]. In this study, ChCl-LA volumes of 5 mL (sample-to-solvent ratio: 1:25), 10 mL (1:50), 15 mL (1:75), and 20 mL (1:100) were investigated. The results are shown in Figure 1D and Figure 2D. In general, increasing the volume of ChCl-LA from 5 mL to 10 mL leads to a significant increase in the content of all individual PC compounds (Figure 2D). More specifically, the highest content of chlorogenic acid was found in the extract obtained with 15 mL of ChCl-LA, while for the other compounds, after increasing the content using 10 mL of the extraction solvent, further addition did not lead to a change in the final concentration (mg/10 g DW). Direct comparison of the results obtained in this study with previously published data is difficult because the volume of solvent used for elution depends on several factors, namely: the physicochemical properties of the solvent, the methods used to accelerate the MSPD extraction process, the extraction conditions, and the type of sample analyzed. For example, when ionic liquids were used as eluents, researchers have reported sample-to-solvent ratios ranging from 1:8 [38] to 1:60 [8] or even 1:125 [17] as optimal. Even for organic solvents, whose physicochemical properties are well known, the results differ from study to study. Indeed, Du et al. [15] have reported a sample-to-solvent ratio of 1:5 as the optimal for the UAE-MSPD extraction of PC from the *Fructus Psoraleae* using 75% EtOH as the extraction solvent. On the other hand, Mansur et al., for the same solvent have determined a ratio of 1:100 as the optimal [40].

In the TPC measurements (Figure 1D), the highest value was found in the extract obtained with 20 mL of ChCl-LA (41.33 ± 0.22 mgGA/g DW). This result can probably be explained by the non-selectivity of the spectrophotometric method for the determination of PC, and consequently, the increase in the volume of the extraction solvent led to the extraction of other compounds with the hydroxyl group in the structure, resulting in an increase in the extraction yield of TPC. In the spectrophotometric determination of antioxidant capacity, we found some deviations from the expected trends in the results (Figure 1D). In fact, it was observed that the FRAP values of the extracts prepared with 15 mL (115.72 ± 5.37 mg Fe^2+^/g DW) and 20 mL (165.85 ± 7.50 mg Fe^2+^/g DW) of NADES were significantly higher than those of the extract prepared with 10 mL of extractant (84.42 ± 1.02 mg Fe^2+^/g DW). This phenomenon can probably be explained by the differences in the final concentration of ChCl- LA in the studied samples, which affect the final result of the antioxidant assay. Indeed, Jurić et al. investigated the antioxidant capacity of pure NADES using the FRAP assay and reported higher FRAP activity of acid-based NADES (ascorbic acid-based NADES) compared to the other tested NADES (polyalcohol-, sugar- and amide-based NADES), which can be attributed to the higher acidity of these solvents [44]. Our results agree well with their findings.

Finally, the highest antioxidant power determined by ABTS assay was observed for the extract prepared with 10 mL of ChCl-LA (23.37 ± 0.76 mg TE/g DW), whereas no differences were observed in all other results (Figure 1D).

### 2.3. Stability Study

**Figure 3:**
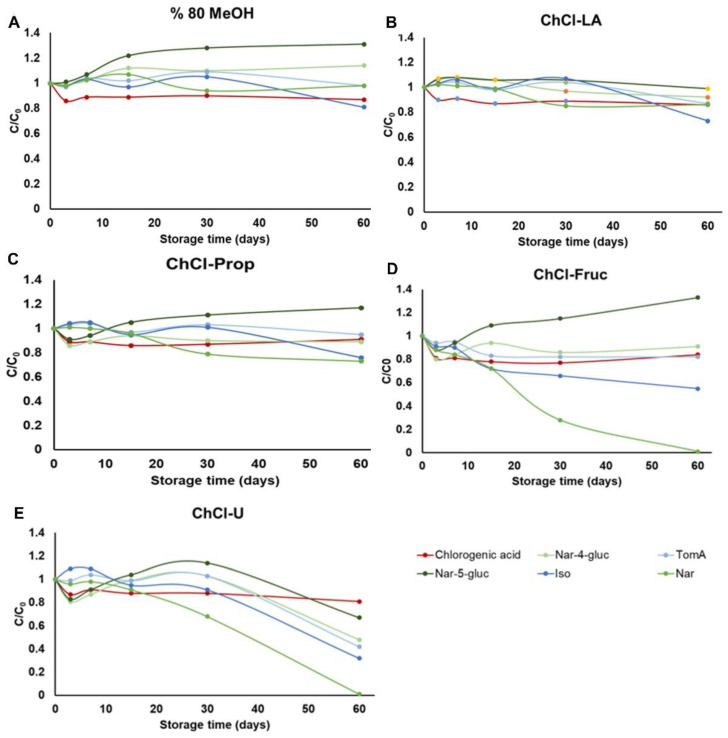
Stability of the selected phenolic compounds extracted from *H. arenarium* with five different solvents (**A**) 80% MeOH, (**B**) choline chloride-lactic acid (1:2) NADES, (**C**) choline chloride:1,2-propanediol (1:2) NADES, (**D**) choline chloride:fructose:water (2:1:1) NADES and (**E**) choline chloride:urea (1:2) NADES), over a period of 0 to 60 days, stored at −18 °C.

**Figure 4:**
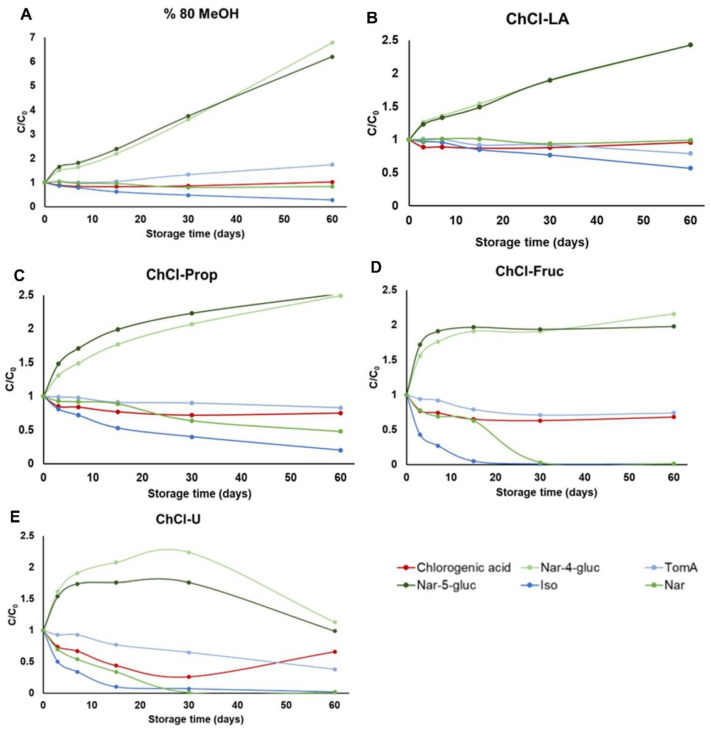
Stability of the selected phenolic compounds extracted from *H. arenarium* with five different solvents (**A**) 80% MeOH, (**B**) choline chloride-lactic acid (1:2) NADES, (**C**) choline chloride:1,2-propanediol (1:2) NADES, (**D**) choline chloride:fructose:water (2:1:1) NADES and (**E**) choline chloride:urea (1:2) NADES), over a period of 0 to 60 days, stored, stored at 4 °C.

**Figure 5:**
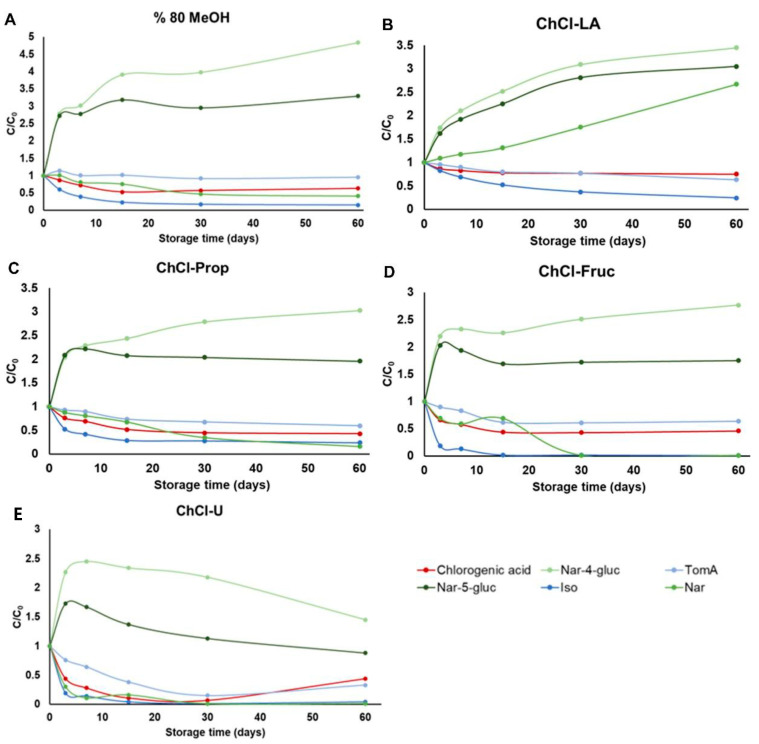
Stability of the selected phenolic compounds extracted from *H. arenarium* with five different solvents (**A**) 80% MeOH, (**B**) choline chloride–lactic acid (1:2) NADES, (**C**) choline chloride:1,2-propanediol (1:2) NADES, (**D**) choline chloride:fructose:water (2:1:1) NADES and (**E**) choline chloride:urea (1:2) NADES), over a period of 0 to 60 days, stored, stored at 25 °C.

PC from natural sources can be effectively used in a variety of industries, including food processing, cosmetics, and pharmaceuticals, where stability is a critical factor in formulating desired products [45]. Due to the physicochemical properties of DES/NADES (mainly low evaporation pressure), the preparation of the dried bioactive plant extracts is difficult [34]. Therefore, new trends are moving towards the direct use of those extracts for the desired purposes [35,36,46]. To date, only few studies have been published on the stability of bioactive compounds in DES/NADES, and most of them focused on anthocyanins [6,45,47,48] and natural dyes, such as curcumin [49,50] and carthamine [4]. Considering these facts, the present study investigated the stability of the main PC from the *H. arenarium* NADES extracts over a period of 0 to 60 days at different temperatures (−18 °C, 4 °C and 25 °C). All the results are graphically presented in Figure 3, Figure 4 and Figure 5 and in the Supplementary material (Figures S2–S4).

From the results presented in Figure 3A–C, it can be concluded that all the studied compounds from the extracts stored in the refrigerator at −18 °C were quite stable when 80% MeOH, ChCl-LA, and ChCl-Prop were used as solvents. On the other hand, in sugar-based NADES (ChCl-Fruc), a loss of 80% of naringenin aglycone (Nar) was observed after one month of extract storage, while the concentration of calcones TomA and Iso decreased by 20% and 40% of the initial concentration after 60 days of storage (Figure 3D). At the same time, the concentration of Nar-5-*O*-gluc also increased by about 40% of the initial value, probably due to the conversion of Iso to naringenin glucoside [51]. Obluchinskaya et al. also demonstrated higher stability of lipophilic compounds from *Fucus vesiculosus* in lactic acid-based NADES compared to glucose-based NADES [48], while on the other hand, higher long-term stability of carthamine in sugar-based NADES compared to organic acid-based NADES was observed in the study by Dai et al. [4]. In the case of urea-based NADES (ChCl-U), a significant decrease in the initial content of all analyzed compounds was observed after 30 days of storage of the extracts, with the exception of chlorogenic acid. Similar results were also observed in the recently published study by Kang et al. who described an accelerated degradation of the compounds from the catechin subclass in the choline chloride–urea-based NADES, in contrast to the thiourea and ascorbic acid-based NADES, which showed stabilizing effects to those compounds [52].

At a storage temperature of 4 °C, the highest stability of all analyzed compounds was generally observed in lactic acid-based NADES (ChCl-LA) (Figure 4B). In the case of the methanolic extract (Figure 4A), a sharp increase in Nar-4-gluc and Nar-5-gluc was observed, which may be attributed to a decrease in the concentrations of TomA, Iso, and Nar with prolongated storage time. A similar trend was also observed for polyalcohol-based NADES (ChCl-Prop, Figure 4C). In contrast, for sugar-based NADES (ChCl-Fruc, Figure 4D), the greatest transformation occurred during the first 7 days of extract storage, among which approximately 70% of the Iso chalcone was lost due to increasing naringenin glucosides concentrations. Moreover, no significant changes were observed in the structure of the extract when the storage time was further increased (from 7 to 60 days). Finally, the most interesting results were found in the case of the ChCl-U extract (Figure 4E). Namely, when the storage time was 30 days, the Iso chalcone and Nar aglycone were completely degraded, while the concentration of naringenin glucosides consequently increased. However, after 30 days of storage of the extract, further degradation of naringenin glucosides also occurs. Based on these results, it is likely that the stability of the selected compounds is influenced not only by the temperature but also by their chemical structures and the pH values of the extraction agents used. As reported by Fu et al. the stability of flavonoids is influenced by the number and position of hydroxyl, glucoside, acyl, and methyl groups in their structures [53]. On the other hand, the relation between the stability of PC and the pH of the solvent has already been confirmed by numerous researchers [54,55]. In a recent study, Liu et al. showed that at a pH higher than 5.0, the content of total flavonoids extracted from sweet potato leaves decreased significantly [56]. Considering that the pH of ChCl-LA was about 1.80, the pH values of polyalcohol-based NADES ranged from 5.70 to 5.90 (depending on the molar ratio between HBA and HBD compounds), urea-based NADES (1:1) had a pH of 4.10, and fructose-based NADES had a pH of 5.8 [44], the different behavior of the selected PC in the tested NADES can be explained. Kang et al., who investigated the stability of epigallocatechin gallate in various NADES, also pointed out that NADES are not in general stabilizing solvents for PC, but that their stabilizing effect is the result of several factors, including interactions between the target components and the solvents themselves [52]. However, due to the synergistic effect between the compounds in the complex extracts as obtained in our study, different transformations are possible, and it is difficult to define a precise degradation/isomerization pathway of each analyte.

Finally, at a temperature of 25 °C, changes in the concentrations of the analyzed compounds and in the composition of the extracts can be observed in all tested solvents after only 7 days of storage (Figure 5). However, the lowest reduction in the initial concentrations of the target compounds was observed in lactic acid-based NADES (ChCl-LA, Figure 5B). The lowest stability was observed in sugar-based NADES (ChCl-Fruc, Figure 5D) and urea-based NADES (ChC-U, Figure 5E). Similar trends can be observed in the stability of the analyzed compounds in 80% MeOH (Figure 5A) and polyalcohol-based NADES (ChCl-Prop, Figure 5C).

## 3. Materials and Methods

### 3.1. Samples and Chemicals

*H. arenarium* inflorescences were kindly provided by the specialized market for dried plant material (Natural Loti, Slovenia). Sample was ground in the electric blender for 1 min, sieved, and kept in a dark place before analysis.

Choline chloride (≥98%), lactic acid (85%), 1,2-propanediol (99%), D-(-)-fructose (≥99%), urea (99–101%), potassium persulfate (K_2_S_2_O_8_, ≥99.5%), iron (III) chloride hexahydrate (FeCl_3_ × 6H_2_O, ≥99%), sodium acetate (CH_3_COONa, p.a.), 2,4,6-tri(2-pyridyl)-s-triazine reagent (TPTZ, ≥99%), 2,2′-azino-bis(3-ethylbenzothiazoline-6-sulfonic acid) diammonium salt (ABTS reagent), (±)-6-hydroxy-2,5,7,8-tetramethylchromane-2-carboxylic acid (Trolox, 97%) as well as the analytical standards: chlorogenic acid (>98.0%) and naringenin (≥98.0%) were acquired from Sigma-Aldrich (St. Louis, MO, USA). The HPLC standard for isosalipurposide (phlorizin) (99.9%) was obtained from ChromaDex (Los Angeles, CA, USA). Folin Ciocalteu’s reagent (FCR), glacial acetic acid (99.8%), anhydrous sodium carbonate (Na_2_CO_3_, >99.5%), iron (II) sulfate heptahydrate (FeSO_4_ × 7H_2_O, ≥99.5%) as well as gallic acid standard (99%) were supplied by Merck (Darmstadt, Germany). HPLC-grade methanol (MeOH) and ethanol (EtOH, 96%) were supplied by Honeywell (Germany) and Kefo (Slovenia), respectively. Ultrapure water was treated in the laboratory on a daily basis.

### 3.2. Preparation of Eutectic Liquids

Four selected natural deep eutectic solvents (NADES): choline chloride:lactic acid (ChCl:LA, molar ratio of 2:1), choline chloride:1,2-propanediol (ChCl:Prop, 1:2), choline chloride:fructose:water (ChCl:Fruc, 2:1:1) and choline chloride:urea (ChCl:U, 1:2) were prepared according to the heating and stirring method previously described [26]. Briefly, the mass of the selected HBA and HBD corresponding to the indicated molar ratio was weighed into an Erlenmeyer flask with a ground stopper. The solid mixtures were stirred magnetically at elevated temperature (80 °C) until colorless transparent liquids were formed (not more than 3 h). Sample was ground in the electric blender for 1 min, sieved, and kept in a dark place before analysis.

### 3.3. Vortex Assisted-Matrix Solid-Phase Dispersion Extraction (VA-MSPD) Procedure

In the preliminary experiments, 200 mg of an accurately weighed *H. arenarium* sample and 500 mg of Florisil RP (Fluka) adsorbent (the ratio of sample to adsorbent was 1:2.5) were placed in an agate mortar. The mixture was ground and homogenized with a pestle for 2 min and quantitively transferred to a 50 mL centrifuge tube. AN amount of 10 mL of eluent (80% MeOH or selected NADES) was added and then vortex shaken thoroughly for 2 min. In addition, the tubes were placed in a laboratory centrifuge (Eppendorf, 5804 R) at 10,000 rpm for 15 min. Clear supernatant was collected, filtered through a 0.45 µm PTFE filter, and used for quantification (spectrophotometric or chromatographic) after appropriate dilution.

### 3.4. Ultrasound-Assisted Extraction (UAE)

For comparison, UAE was performed with 80% MeOH according to our recently published work [26]. Briefly, 500 mg of the ground *H. arenarium* sample was weighed into a 50 mL conical centrifuge tube and 15 mL of 80% MeOH was added. The mixture was sonicated at an elevated temperature of 50 °C for 1 h. After centrifugation at 11,000 rpm for 15 min, the clear supernatant was decanted, and the extraction was repeated a second time with 10 mL of fresh 80% MeOH. The supernatant from the second extraction cycle was combined with the first in the 25 mL volumetric flask and additionally pooled with the 80% MeOH to the mark.

### 3.5. Optimization of VA-MSPD Conditions

Several factors can significantly affect the efficiency of VA-MSPD extraction. The most important are: type of adsorbent, type and volume of eluent, ratio of sample to adsorbent (S/A ratio), grinding time, and vortex time [8,15,17]. Consequently, the influence of these parameters on the proposed VA-MSPD method for the extraction of PC from *H. arenarium* using the one-factor-at-a-time (OFAT) method was investigated in detail. All optimization experiments were performed in duplicate:

(a) After selecting the most effective extraction solvent based on the results of the preliminary extraction experiment, four different adsorbents, including: Florisil RP (0.15–0.25 mm, 60–100 mesh ASTM, Fluka), C18 Silica gel spherical (0.7–0.9 cm^3^/g pore volume, 200–400 mesh, Supelco), and silica with two different pore sizes; silica 60 (0.2–0.5 mm, 35–70 mesh ASTM, Fluka) and silica (0.063–0.20 mm, 70–230 mesh ASTM, Kemika), were tested. Other factors were as follows: S/A ratio of 1:2.5 (200 mg sample and 500 mg of the stationary phase), 2 min grinding and vortex time, and 10 mL of the selected NADES.
(b) In the second phase, after selecting the most effective adsorbent, four different S/A ratios (1:0.5, 1:1, 1:1.5, and 1:2) were evaluated. For comparison, a sample extraction without addition of adsorbent was also performed. Other parameters were: 2 min grinding and vortex time and 10 mL of the selected NADES.
(c) Vortex time was studied in a range of 1 to 4 min, while the other parameters were kept constant: S/A ratio determined from the results of experiments (a) and (b), grinding time of 2 min and 10 mL of the selected NADES.
(d) Finally, the volumes of the selected NADES (5 mL–20 mL) were examined. Other extraction conditions were: S/A ratio and vortex time based on the results of the previous experiments and 2 min grinding time

### 3.6. Stability Study of the Obtained VA-MSPD Extracts

The storage stability of *H. arenarium* extracts prepared with 80% MeOH and/or the four NADES used (ChCl:LA, ChCl:Prop, ChCl:Fruc, ChCl:U), prepared during preliminary experiments, was monitored according to the procedures described in previously published studies [4,6]. Briefly, the extracts were stored during the 60 days under different conditions: (a) in the freezer at −18 °C; (b) in the refrigerator at 4 °C; and (c) under room conditions at 25 °C. After a specified time (3 days, 7 days, 15 days, 1 month, and 2 months), the extracts were properly diluted with 80% MeOH and analyzed by described HPLC-UV method (Section 3.7.4). The results were plotted graphically as the rate of degradation (C/C_0_) as a function of time; where C_0_ is the initial concentration of the selected compound and C is the concentration after the selected storage time.

### 3.7. Analytical Procedures

#### 3.7.1. Total Phenolic Content (TPC)

Total phenolic content (TPC) was determined by the standard spectrophotometric method [57]. Briefly, 40 µL of the properly diluted extracts (VA-MSPD and UAE) were mixed with 3.160 mL of ultrapure water and 200 µL of diluted Folin–Ciocalteu’s reagent (20%, *V:V*). After 7 min, 600 µL of 20% Na_2_CO_3_ was added, and the mixture was shaken and stored in a dark place for 2 h. Absorbance at 765 nm was measured and results were expressed as mg gallic acid equivalent per g dry weight (mg GA/g DW). The gallic acid calibration curve (*R*^2^ = 0.9998, y = 0.0008x + 0.004) was plotted in a concentration range of 25 mg/L to 250 mg/L.

#### 3.7.2. ABTS Radical Scavenging Assay

The radical scavenging capacity of the VA-MSPD extracts, expressed as mg Trolox equivalent per gram dry weight (mg TE/g DW), was determined using the standard ABTS assay with some modifications [58]. Briefly, the cationic ABTS radical reagent (ABTS^•+^) was prepared (16 h before use) by mixing equal volumes of a 7 mM solution of ABTS and a 2.4 mM solution of K_2_S_2_O_8_. The absorbance of the reagent was adjusted to pH 0.70 ± 0.05 at 734 nm by dilution with 96% EtOH. For the measurements, 3950 µL of the prepared ABTS^•+^ was mixed with 50 µL of Trolox standard solution (in the concentration range of 0.1–1 mM) or appropriately diluted *H. arenarium* extracts. The scavenging effect, expressed in %, was calculated using the following equation:

(1) $$\mathrm{Scavenging}\;\mathrm{effect}\;\left(\%\right)=\frac{\left(\mathrm{AB}-\mathrm{AA}\right)}{\mathrm{AB}}\times 100$$

where AB represents the absorbance of the mixture of ABTS^•+^ and EtOH (blank value), while AA represents the absorbance of the mixture of ABTS^•+^ and extracts/Trolox solution.

#### 3.7.3. FRAP Assay

The antioxidant capacity of the VA-MSPD extracts, expressed as mg Fe^2+^ ion equivalent per gram dry weight (mg Fe^2+^/g DW), was determined by a standard method with slight modifications [59]. In short, the daily fresh reagent FRAP was prepared by mixing acetate buffer (pH = 3.60), 10 mM TPTZ solution (prepared in 40 mM HCl), and 20 mM FeCl_3_x6H_2_O in a 10:1:1 ratio at 37 °C [60]. Working solutions of Fe^2+^ ions in the concentration range of 10–300 mg/L were prepared by diluting 20 mM Fe^2+^ (FeSO_4_ × 7H_2_O) standard solution with ultrapure water. For measurements, 4950 µL of the FRAP reagent thus prepared was mixed with 50 µL of Fe^2+^ working solutions or appropriately diluted crude extracts. After 30 min incubation at 37 °C, the absorbances were measured at 593 nm against FRAP reagent as blank.

#### 3.7.4. Quantification of Individual Phenolic Compounds by High-Performance Liquid Chromatography with UV Detection (HPLC-UV)

For chromatographic analysis of the six most dominant PC identified in *H. arenarium* [23,61,62], the previously developed and validated HPLC-UV method was applied [26]. Briefly, a Varian (ProStar) chromatography system was used in combination with an Eclipse XDB–C18 column (150 mm × 4.6 mm i.d., 5 μm) from Agilent (Santa Clara, CA, USA). The mobile phase consisted of acetonitrile (eluent A) and water with 1% (*v*/*v*) acetic acid (eluent B) using gradient elution (0–1 min 100% B, 1–5 min 100–90% B, 5–30 min 90–59% B, 30–31 min 100%) at room temperature (RT) and a flow rate of 1 mL/min. The column was re-equilibrated for 10 min at initial conditions between each successive injection. The acquisition wavelength was set to 280 nm. Calibration curves of three external standards (chlorogenic acid, isosalipurposide, and naringenin) prepared in 80% MeOH in the concentration range of 2.5 mg/L to 50 mg/L were constructed.

### 3.8. Statistical Analysis

All extraction experiments were performed in duplicate, and experimental analysis of each parallel sample was performed in triplicate. Results were expressed as mean ± standard deviation and calculated in Excel. A one-way analysis (ANOVA), followed by a Student–Newman–Keuls (S-N-K) post hoc test, was performed using SPSS software (IBM SPSS Statistics for Windows, version 22.0 (Armonk, NY: IBM Corp.)) to determine statistically significant differences between results.

## 4. Conclusions

To improve traditionally used extraction techniques for the isolation of valuable bioactive constituents of the inflorescences of *H. arenarium*, a simple, rapid, and cost-effective vortex-assisted matrix solid-phase dispersion method (VA-MSPD) was proposed. The main advantage of the VA-MSPD developed and optimized in this study is the use of a natural deep eutectic solvent (NADES) as an eco-friendly eluent instead of conventionally used organic solvents. Under the optimal extraction conditions determined, the TPC value of the prepared extract VA-MSPD was 27% higher than that of the methanolic UAE extract, with a 12% increase in the sum of individual phenolic compounds (PC).

Moreover, to the best of our knowledge, this is one of the first reports on the stability of selected PC (chlorogenic acid, naringenin-4′-*O*-glucoside, tomoroside A, naringenin-5-*O*-glucoside, isosalipurposide, and naringenin) in the increasingly used NADES compared to methanolic extract. In general, we confirmed the highest stability of all tested extracts under frozen storage conditions (−18 °C), while light and room temperature (25 °C) accelerated the isomerization/degradation of the investigated compounds. However, the results of this study also suggest a potential stabilizing effect of lactic acid-based NADES under more “aggressive“ conditions (higher temperature and daylight) on the selected PC, which could be of particular importance for the direct use of the obtained NADES plant extracts without further purification (for example in industries where the use of hazardous organic solvents is strongly discouraged, such as in the food, cosmetics, and pharmaceutical industries).

## Figures and Tables

**Table 1:** Influence of NADES composition on VA-MSPD extraction efficiency of total phenolic content (TPC, mg GA/g DW) and major individual phenolic compounds (mg/10 mg DW) from *H. arenarium* inflorescences. Comparison with the reference method UAE.

Method	Solvent	TPC	Chlorogenic Acid	Nar-4-gluc	TomA	Nar-5-gluc	Iso	Nar
		mg GA/g DW	mg/10 g DW					
VA-MSPD	ChCl-LA	38.34 ± 0.09 ^d^	48.99 ± 5.57 ^a^	9.72 ± 0.17 ^b^	19.69 ± 0.45 ^c^	23.33 ± 0.59 ^b^	36.23 ± 0.87 ^d^	8.54 ± 0.29 ^e^
	ChCl-Prop	31.06 ± 0.57 ^c^	38.94 ± 4.09 ^a^	12.40 ± 0.42 ^d^	17.50 ± 0.66 ^b^	30.10 ± 1.09 ^c^	30.87 ± 0.27 ^c^	7.49 ± 0.01 ^c^
	ChCl-Fruc	13.58 ± 2.60 ^a^	37.87 ± 1.54 ^a^	6.96 ± 0.40 ^a^	8.39 ± 0.46 ^a^	16.16 ± 0.70 ^a^	10.44 ± 0.44 ^a^	3.21 ± 0.11 ^a^
	ChCl-U	26.88 ± 0.88 ^b^	38.98 ± 0.13 ^a^	13.38 ± 0.43 ^d^	16.41 ± 0.27 ^b^	32.30 ± 0.70 ^d^	18.00 ± 0.30 ^b^	6.55 ± 0.05 ^b^
	80% MeOH	30.13 ± 0.12 ^c^	43.10 ± 1.79 ^a^	12.09 ± 0.04 ^e^	21.75 ± 0.07 ^d^	28.60 ± 0.30 ^c^	35.99 ± 0.01 ^d^	9.38 ± 0.16 ^f^
UAE	80% MeOH	32.73 ± 0.52 ^c^	43.10 ± 2.69 ^a^	11.19 ± 0.13 ^c^	15.74 ± 1.16 ^b^	28.16 ± 1.44 ^c^	30.68 ± 0.53 ^c^	7.97 ± 0.24 ^d^

Different superscripts for each compound in the same row denoted significant differences between the solvents tested with 95% confidence level (*p* < 0.05) according to the S-N-K test.

## Data Availability

Data for this article can be found online (Supplementary File).

## Supplementary Materials

The following supporting information can be downloaded at: https://www.mdpi.com/article/10.3390/plants11243468/s1, Figure S1: Typical HPLC-UV chromatogram of *H. arenarium* extract. The following compounds were identified: 2. chlorogenic acid; 5. 1,3-dicaffeoylquinic acid; 7. naringenin-4′-O-glucoside; 8. tomoroside A; 9. naringenin-5-O-glucoside; 13. 1,5-dicaffeoylquinic acid; 14. 3,5-dicaffeoylquinic acid; 15. kaempferol 3-O-glucoside; 17. apigenin-7-O-glucoside; 19. 4,5-O-dicaffeoylquinic acid; 20. Isosalipurposide; 23. naringenin; 24. apigenin.; Figure S2: Influence of the extraction solvent on the stability of 5 selected phenolic compounds from *H. arenarium*, over a period of 0 to 60 days, stored at −18 °C: (a) chlorogenic acid; (b) naringenin-4′-O-glucoside; (c) tomoroside A; (d) naringenin-5-O-glucoside; (e) isosalipurposide; (f) naringenin; Figure S3: Influence of the extraction solvent on the stability of 5 selected phenolic compounds from *H. arenarium*, over a period of 0 to 60 days, stored at 4 °C: (a) chlorogenic acid; (b) naringenin-4′-O-glucoside; (c) tomoroside A; (d) naringenin-5-O-glucoside; (e) isosalipurposide; (f) naringenin; Figure S4: Influence of the extraction solvent on the stability of 5 selected phenolic compounds from *H. arenarium*, over a period of 0 to 60 days, stored at 25 °C: (a) chlorogenic acid; (b) naringenin-4′-O-glucoside; (c) tomoroside A; (d) naringenin-5-O-glucoside; (e) isosalipurposide; (f) naringenin.
