# Supplementary material for: Natural Deep Eutectic Solvent-Based Matrix Solid Phase Dispersion (MSPD) Extraction for Determination of Bioactive Compounds from Sandy Everlasting (Helichrysum arenarium L.): A Case of Stability Study
Source: Plants (Basel). 2022 Dec 11;11(24):3468. doi: 10.3390/plants11243468 (PMC9782231; doi:10.3390/plants11243468)
Supplement: Supplementary file 1 [file plants-11-03468-s001.zip › plants-2059293-supplementary.pdf]

SUPPLEMENTARY MATERIAL

Natural deep eutectic solvent-based matrix solid phase dispersion (MSPD) extraction for determination of bioactive compounds from sandy everlasting (*Helichrysum Arenarium* L.): a case of stability study

Milena Ivanović[a], Peter Krajnc[a], Aleš Mlinarič[b], Maša Islamčević Razboršek[a*]

[a]*University of Maribor, Faculty of Chemistry and Chemical Engineering, Smetanova ulica 17, SI-2000 Maribor, Slovenia*

[b] *MARIFARM, proizvodnja in storitve d.o.o, Minařikova ulica 8, SI-2000 Maribor, Slovenia*

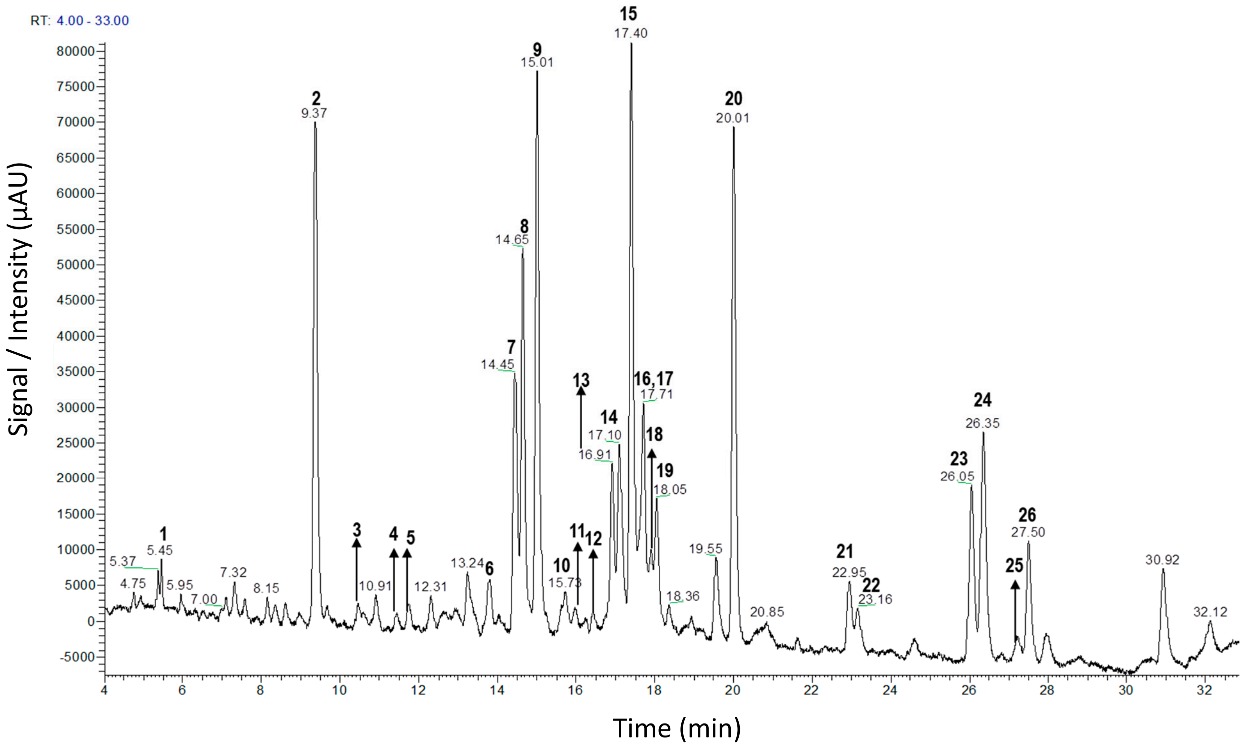

Figure S1. Typical HPLC-UV chromatogram of *H. arenarium* extract . The following compounds were identified: 2. chlorogenic acid; 5. 1,3-dicaffeoylquinic acid; 7.naringenin-4′-*O*-glucoside; 8. tomoroside A; 9. naringenin-5-*O*-glucoside; 13. 1,5-dicaffeoylquinic acid; 14. 3,5-dicaffeoylquinic acid; 15. kaempferol 3-*O*-glucoside; 17. apigenin-7-*O*-glucoside; 19. 4,5-O-dicaffeoylquinic acid; 20. Isosalipurposide; 23. naringenin; 24. apigenin. Identification based on the (Ivanović et al., 2020), Ivanović, M., Albreht, A., Krajnc, P., Vovk, I., & Islamčević Razboršek, M. (2020). Sustainable ultrasound-assisted extraction of valuable phenolics from inflorescences of Helichrysum arenarium L. using natural deep eutectic solvents. *Industrial Crops and Products*, *May*, 113102. https://doi.org/10.1016/j.indcrop.2020.113102

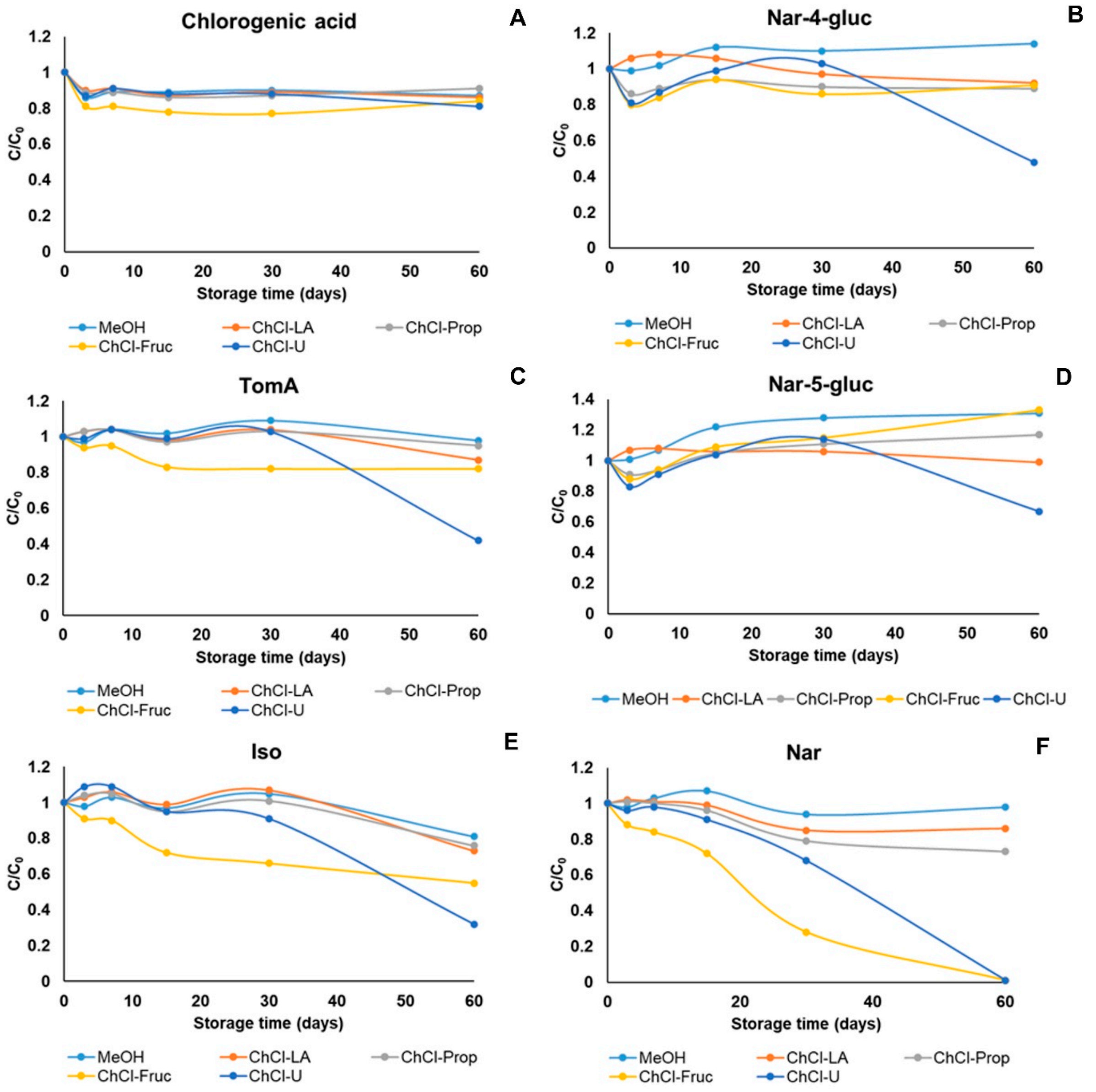

Figure S2. Influence of the extraction solvent on the stability of 5 selected phenolic compounds from *H.arenarium*, over a period of 0 to 60 days, stored at -18 °C: (A) chlorogenic acid; (B) naringenin-4′-*O*-glucoside; (C) tomoroside A; (D) naringenin-5-*O*-glucoside; (E) isosalipurposide; (F) naringenin.

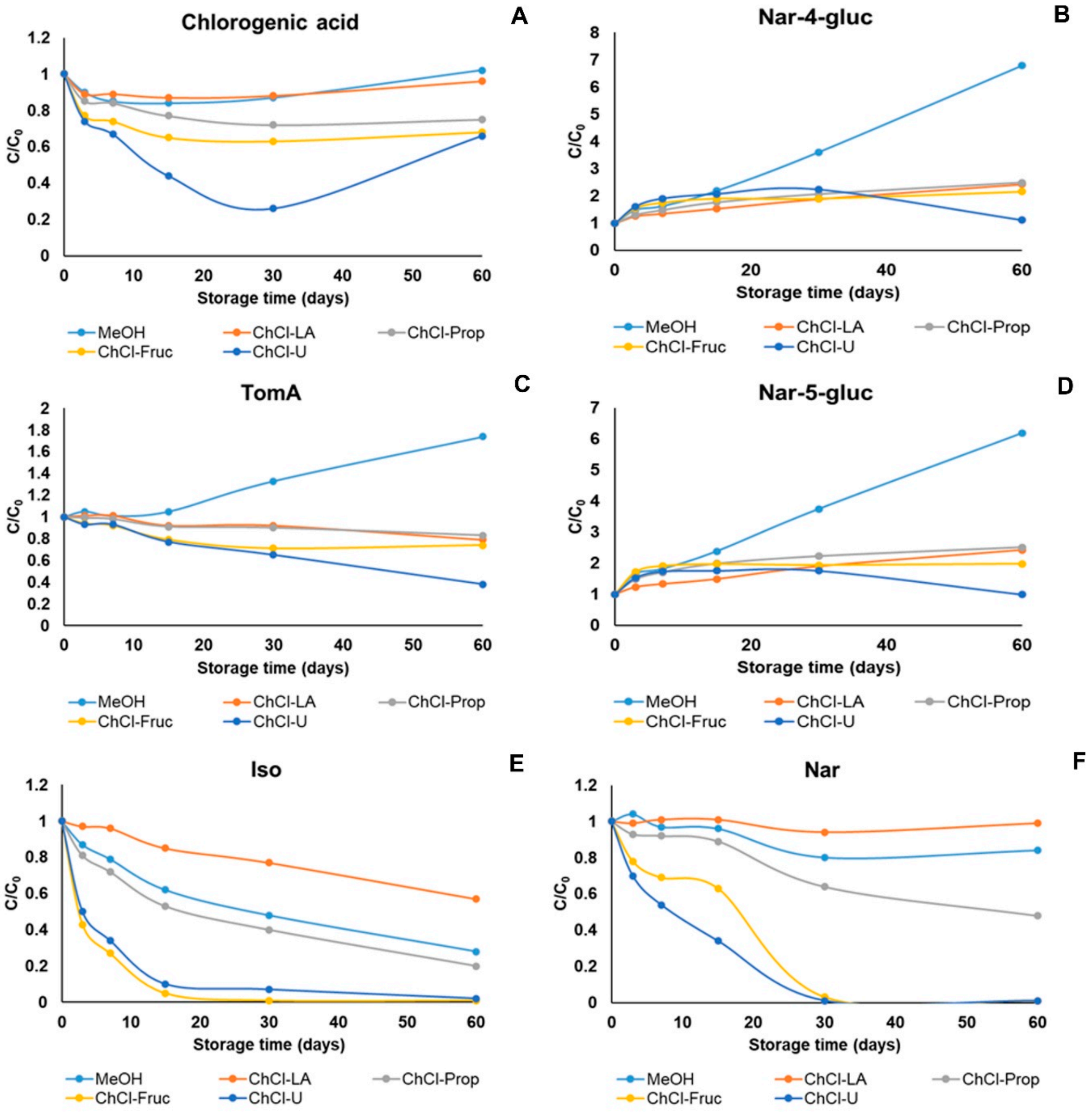

Figure S3. Influence of the extraction solvent on the stability of 5 selected phenolic compounds from *H.arenarium*, over a period of 0 to 60 days, stored at 4 °C: (A) chlorogenic acid; (B) naringenin-4′-*O*-glucoside; (C) tomoroside A; (D) naringenin-5-*O*-glucoside; (E) isosalipurposide; (F) naringenin.

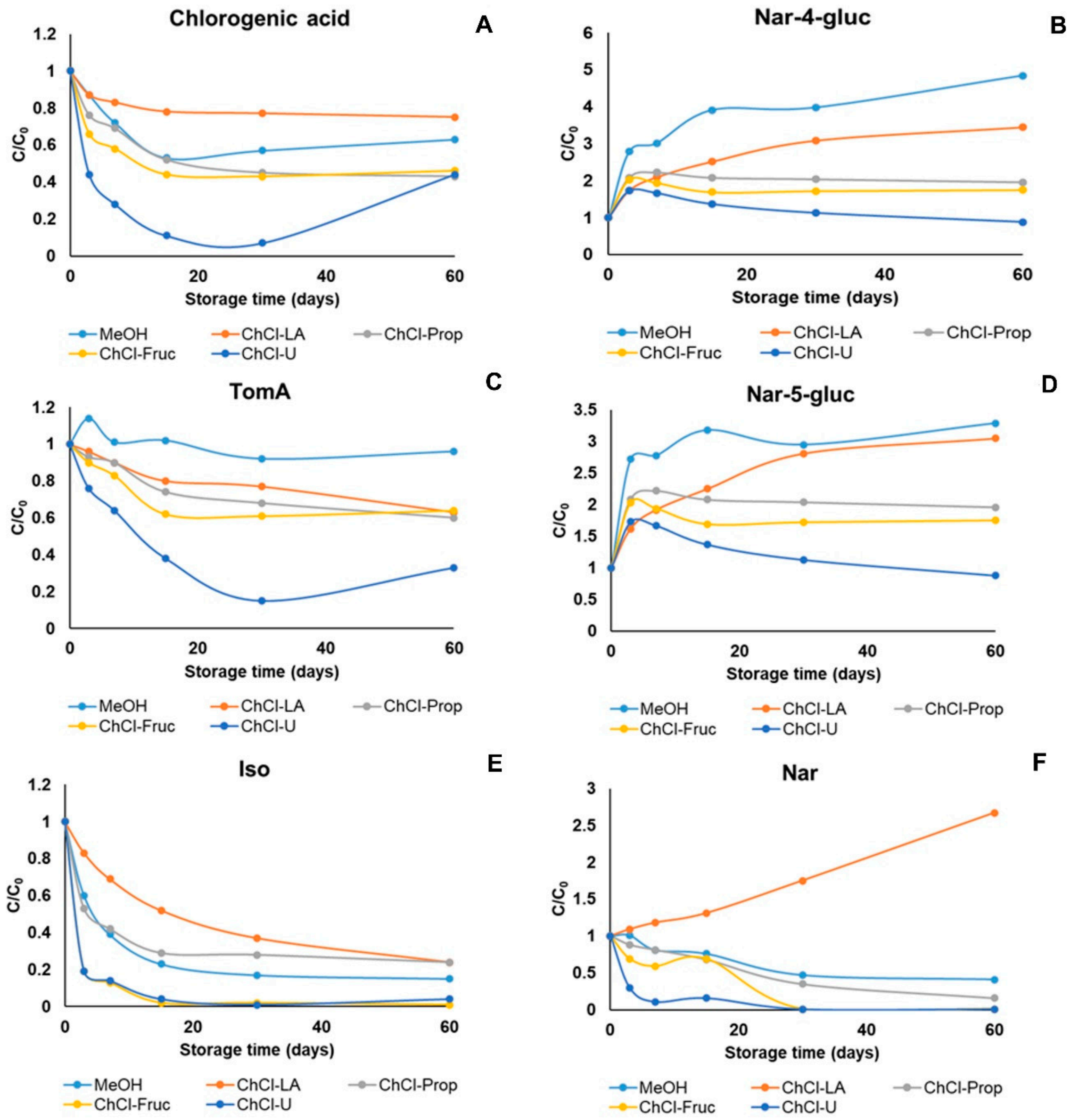

Figure S4. Influence of the extraction solvent on the stability of 5 selected phenolic compounds from *H.arenarium*, over a period of 0 to 60 days, stored at 25 °C: (A) chlorogenic acid; (B) naringenin-4′-*O*-glucoside; (C) tomoroside A; (D) naringenin-5-*O*-glucoside; (E) isosalipurposide; (F) naringenin.
